# Vector competence of mosquitoes from Europe for Tahyna virus

**DOI:** 10.1038/s41598-025-10883-5

**Published:** 2025-07-11

**Authors:** Patrick Höller, Renke Lühken, Felix Gregor Sauer, Carmen Villacañas de Castro, Norbert Becker, Hanna Jöst, Wolf Peter Pfitzner, Jonas Schmidt-Chanasit, Anna Heitmann, Stephanie Jansen

**Affiliations:** 1 Bernhard Nocht Institute for Tropical Medicine, Bernhard-Nocht-Strasse 74, 20359 Hamburg, Germany; 2 Carl von Ossietzky University, Oldenburg, Germany; 3 Institute for Dipterology (IfD), Speyer, Germany; 4 Center for Organismal Studies (COS), University of Heidelberg, Heidelberg, Germany; 5 Kommunale Aktionsgemeinschaft zur Bekämpfung der Schnakenplage e.V, Speyer, Germany; 6 Faculty of Mathematics, Informatics and Natural Sciences, University of Hamburg, Hamburg, Germany

**Keywords:** *Aedes albopictus*, *Aedes rusticus*, Tahyna virus, Vector competence, Viral transmission, Virus-host interactions, Entomology

## Abstract

Tahyna virus (TAHV) was the first mosquito-borne virus isolated in Europe, and has since been found throughout Eurasia and Africa. Infections are mostly asymptomatic but can cause “Valtice fever”, characterized by influenza-like symptoms, mainly in children, with severe cases occasionally causing neurological symptoms. The virus is maintained in an enzootic cycle between small mammals and mosquitoes. Recent and comprehensive studies of vector competence for TAHV are scarce. To fill this gap, and to better understand the transmission cycle of TAHV, we studied ten taxa (*Ae. aegypti*,* Ae. albopictus*,* Ae. japonicus japonicus*,* Ae. koreicus*,* Ae. rusticus*,* Cx. pipiens* biotype *pipiens*,* Cx. torrentium*,* Cs. morsitans/fumipennis*,* An. daciae*, and *An. stephensi*) by orally infecting them with TAHV. All taxa were susceptible to TAHV infection. Additionally, the invasive species *Ae. albopictus*, and *Ae. rusticus*, a species native to Europe, were able to transmit the virus at 27 ± 5 °C, with transmission efficiencies of 3.3% and 14.3%, respectively. Therefore, it is plausible for TAHV to be transmitted by *Ae. albopictus* and *Ae. rusticus* in natural settings in Europe. At a lower temperature of 24 ± 5 °C, infection and transmission decreased in *Ae. albopictus*. This data will allow future risk models and early warning systems to better predict TAHV transmission.

## Introduction

In the last decades, the global health significance of arthropod-borne viruses (arboviruses) has increased dramatically. Recent outbreaks of dengue, chikungunya, Rift Valley fever and Zika viruses have highlighted the threat these viruses pose to human and animal health. The health impact of arboviruses is expected to increase in the future due to globalization and urbanization^1,2^. Tahyna virus (TAHV) was the first identified mosquito-borne virus in Europe, isolated from *Aedes vexans* in 1958 in former Czechoslovakia^3^. TAHV is a member of the genus *Orthobunyavirus*, within the *Peribunyaviridae* family^4^. All members of this genus are arthropod-borne, besides biting midges or ticks, different mosquito species are considered the main vector. The genus is further subdivided into 18 different serogroups according to the virus’ genetic and antigenic relationship. TAHV belongs to the California serogroup, named after the California encephalitis virus^5^. Like all members of the *Peribunyaviridae* family, TAHV is enveloped and has a tri-segmented negative-sense RNA genome, showcasing remarkable conservation^4,6^. *Orthobunyaviruses* can exchange those genome segments during coinfection, a process called reassortment which can potentially lead to heightened pathogenicity^7^. Reassortment has been observed between La Crosse virus and TAHV in *Aedes triseriatus* in laboratory settings^8^. As TAHV co-circulates with other California serogroup viruses, studying its vector competence is crucial to assess potential reassortment risks.

TAHV is maintained in an enzootic cycle. Based on laboratory infection, and serological studies, the most likely amplifying host is the European hare^9^. However, many different mammals, including non-human primates, have shown viremia and seroconversion in laboratory studies^10^. Antibodies to TAHV have also been detected in different wildlife and livestock species, including deer, wild boars, mouflons, rodents, sheep, horses, cattle, and pigs, but no symptoms have been reported in these species^10,11^. Historically, hedgehogs were thought to be a possible overwintering reservoir for the virus, however, these findings are being questioned, and the true role of hedgehogs in the transmission cycle remains unclear^9,11,12^. TAHV causes a human disease, known as “Valtice fever”, first documented in the 1960s^13,14^. The majority of infections are asymptomatic. Symptoms are observed mainly in children in late summer and early autumn. The disease presents with flu-like symptoms, myalgia, and other symptoms typical for California serogroup viruses, including headache, nausea, conjunctivitis, stiff neck, and can in rare cases cause meningitis and encephalitis^10^. It has been shown in mice that the degree of neuroinvasiveness varies depending on the strain, however, no data on the medical relevance of different TAHV strains is available^6^. To this day, no fatalities have been recorded^10^. As it is not a notifiable disease and the symptoms are not specific, the number of people affected is thought to be drastically under-reported. While TAHV is predominantly found in Europe, there have also been detections of TAHV in vertebrates and mosquitoes in Asia (including Russia) and Africa^15^. However, reports from African countries primarily rely on serology, therefore, raising the possibility that the findings of TAHV are actually due to cross-reactivity with closely related California serogroup viruses, such as Lumbo virus^10^. Focusing on the situation in Europe, studies from the 1960s in former Czechoslovakia reported high TAHV seroprevalence, reaching 80–90% among elderly residents in endemic areas and up to 30% in regions with mass mosquito occurrence^14,16,17^. More recently, 10% of patients with unsolved neuroinvasive disease in Croatia between 2017 and 2022, and 0.3% of blood donors in the Alps (Austria, Italy) in 2014 tested positive for TAHV antibodies^18,19^. TAHV has not been detected in Germany in recent years, and the reasons for its absence remain unclear. One possible explanation is the lack of surveillance for this arbovirus, meaning the virus could be circulating at undetected levels. The lack of clarity about which mosquito species transmit the virus complicates targeted monitoring efforts.

After its first isolation from *Ae. vexans*, TAHV has since been identified in various species of the genera *Aedes*, *Culex*, *Culiseta*, and *Anopheles*^10,17,19–24^. However, studies for vector competence are rare. Differentiating between the detection of viruses in field-collected specimens and the species’ ability to transmit the virus by excreting infectious viral particles in their saliva is crucial. Only the latter can serve as direct evidence for vector competence of a particular mosquito species towards a certain virus. A European study assessed vector competence for *Ae. vexans*, which was shown to be capable of transmitting the virus^25^. In a separate study conducted in China, both *Ae. albopictus* and *Cx. pipiens pallens* were tested, however, only *Ae. albopictus* was found to transmit the virus^26^. Furthermore, it has been proven that *Ae. vexans* can transmit TAHV transovarially to its offsprings^27^. Overwintering of TAHV-infected mosquitoes has been shown for *Cs. annulata* adults as well as *Ae. vexans* eggs^22,27^.

To address the gap of knowledge concerning the vector competence of mosquitoes for TAHV we conducted this study with a diverse range of mosquito taxa, including both those native and exotic in Europe. We also investigated the effect of temperature on the risk of transmission by *Ae. albopictus.* The results of this study will help to understand the transmission cycle of TAHV.

## Results

### Feeding and survival rates

Feeding rates (number of engorged mosquitoes per number of mosquitoes offered the blood meal) varied between 18.7% and 71.4% among all taxa, with no consistent pattern observed across genera (Table 1). Survival rates (number of alive mosquitoes after incubation period per number of engorged mosquitoes) were generally high (Table 1), except for *An. stephensi* and *Cs. morsitans/fumipennis* which exhibited a higher mortality rate. Given the limited availability of *Cs. morsitans/fumipennis*, the incubation period for these specimens was shortened to seven days.

**Table 1:** Total input number of mosquitoes offered a blood meal per taxon and temperature. Feeding rate: number of engorged mosquitoes per total input. Survival rate: number of alive mosquitoes after incubation period per number of engorged mosquitoes.

Mosquito taxon	Incubation period [days]	Temperature [°C]	Total number of mosquitoes	Feeding rate [%]	Survival rate [%]
*Ae. aegypti*	14	27 ± 5	187	18.7 (35/187)	91.4 (32/35)
*Ae. albopictus*	14	24 ± 5	88	54.5 (48/88)	81.3 (39/48)
	14	27 ± 5	85	50.6 (43/85)	79.1 (34/43)
*Ae. j. japonicus*	14	27 ± 5	190	25.3 (48/190)	70.8 (34/48)
*Ae. koreicus*	14	27 ± 5	7	71.4 (5/7)	100 (5/5)
*Ae. rusticus*	14	27 ± 5	12	66.7 (8/12)	87.5 (7/8)
*Cx. pipiens* biotype *pipiens*	14	27 ± 5	73	63.0 (46/73)	95.7 (44/46)
*Cx. torrentium*	14	27 ± 5	84	41.7 (35/84)	91.4 (32/35)
*Cs. morsitans*/*fumipennis*	7	27 ± 5	31	48.4 (15/31)	33.3 (5/15)
*An. daciae*	14	27 ± 5	2	50.0 (1/2)	100 (1/1)
*An. stephensi*	14	27 ± 5	175	29.7 (52/175)	51.9 (27/52)

### Infection and transmission

**Fig. 1:**
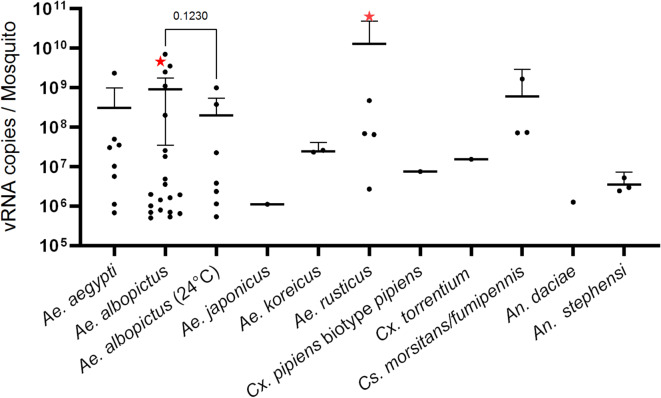
The concentration of viral RNA copies per mosquito body at 27 °C, unless indicated otherwise. Only data above the limit of detection is shown. Dots represent single mosquito specimens. The mean and 95% confidence interval are shown. Red stars indicate specimens with a corresponding positive saliva.

TAHV was able to infect all of the tested taxa, with varying infection rates (Table 2). The lowest infection rates were observed in both species of the genus *Culex*, and in the species *Ae. j. japonicus*, with 3.3% of specimens testing positive for TAHV RNA (Table 2). *Aedes albopictus* incubated at 27 °C and *Ae. rusticus* displayed the highest infection rates among all taxa, with 70.0% and 71.4% respectively (Table 2). *Anopheles daciae* was excluded from this comparison due to low sample size. *Aedes albopictus* and *Ae. rusticus* were also the only ones able to transmit TAHV, with transmission efficiencies of 3.3% for *Ae. albopictus* and 14.3% for *Ae. rusticus* at 27 °C. Additionally, these two species showed the highest mean number of viral RNA copies per mosquito body, with 9.05 × 10^8^ and 1.29 × 10^10^ respectively (Fig. 1; Table 2). Transmission efficiency was assessed by salivation and cell culture followed by RT-qPCR on cell supernatant of positive samples. For *Ae. rusticus*, the specimen with the highest body titer was also the one with the positive saliva sample. In contrast, multiple *Ae. albopictus* specimens had similar or even higher body titers than the one with a positive saliva, yet none of these specimens had viral particles in their saliva (Fig. 1).

To investigate the potential influence of temperature on the infectivity and transmission of TAHV, an additional experiment with *Ae. albopictus* was conducted at an incubation temperature of 24 °C. The results revealed that reducing the mean incubation temperature by 3 °C, significantly decreased the infection rate from 70.0 to 23.3% (Fisher’s exact test; *p* = 0.0006) (Table 2). In addition to the reduced infection rate, the transmission efficiency also decreased from 3.3 to 0.0% (Fisher’s exact test; *p* > 0.9999), indicating that no transmission occurred at the lower temperature. The mean body titer also decreased from 9.05 × 10^8^ to 2.00 × 10^8^ (Welch’s test; *p* = 0.1230) (Fig. 1; Table 2). However, neither of these changes reached statistical significance. During the salivation assay of *An. stephensi*, two specimens were damaged beyond usability, resulting in a lower number of tested individuals (*n* = 25; Table 2) compared to the number of live mosquitoes initially available (*n* = 27; Table 1).

**Table 2:** Vector competence studies per taxon and temperature. Infection rate: number of positive bodies per total number of tested mosquitoes. Transmission efficiency: number of positive salivas per total number of tested mosquitoes. The mean body titer is given as the mean number of viral RNA copies per mosquito body, with 95% confidence interval. Na: Non applicable, confidence interval could not be calculated due to a singular positve body sample.

Mosquito taxon	Temperature [°C]	Number of tested mosquitoes	Infection rate [%]	Transmission efficiency [%]	Mean body titer as vRNA copies per mosquito (95% confidence interval)
*Ae. aegypti*	27 ± 5	30	26.7 (8/30)	0.0 (0/30)	3.09 × 10^8^ (0, 9.95 × 10^8^)
*Ae. albopictus*	24 ± 5	30	23.3 (7/30)	0.0 (0/30)	2.00 × 10^8^ (0, 5.48 × 10^8^)
	27 ± 5	30	70.0 (21/30)	3.3 (1/30)	9.05 × 10^8^ (3.47 × 10^7^, 1.78 × 10^9^)
*Ae. j. japonicus*	27 ± 5	30	3.3 (1/30)	0.0 (0/30)	1.12 × 10^6^ (na)
*Ae. koreicus*	27 ± 5	5	40.0 (2/5)	0.0 (0/5)	2.46 × 10^7^ (8.08 × 10^6^, 4.11 × 10^7^)
*Ae. rusticus*	27 ± 5	7	71.4 (5/7)	14.3 (1/7)	1.29 × 10^10^ (0, 4.82 × 10^10^)
*Cx. pipiens* biotype *pipiens*	27 ± 5	30	3.3 (1/30)	0.0 (0/30)	7.54 × 10^6^ (na)
*Cx. torrentium*	27 ± 5	30	3.3 (1/30)	0.0 (0/30)	1.54 × 10^7^ (na)
*Cs. morsitans*/*fumipennis*	27 ± 5	5	60.0 (3/5)	0.0 (0/5)	6.05 × 10^8^ (0, 2.90 × 10^9^)
*An. daciae*	27 ± 5	1	100 (1/1)	0.0 (0/1)	1.26 × 10^6^ (na)
*An. stephensi*	27 ± 5	25	12.0 (3/25)	0.0 (0/25)	3.54 × 10^6^ (0, 7.23 × 10^6^)

## Discussion

In this study, we found that all ten tested mosquito taxa of the genera *Aedes*,* Culex*,* Culiseta*, and *Anopheles* were susceptible to TAHV infection. *Aedes albopictus* and *Ae*. *rusticus* were shown to be able to transmit the virus at an efficiency of 3.3% and 14.3%, respectively. Notably, both of the species belong to the *Aedes* genus. This aligns with previous findings on *Ae. vexans*, the only other mosquito species from Europe confirmed as a competent vector for TAHV in laboratory studies^25^. Additionally, several other *Aedes* species, such as *Ae. caspius* and *Ae. sticticus*, are believed to play important roles in the transmission cycle, as TAHV has been detected in field-collected specimens^10,28^.

Vector competence is an important but not exclusive factor to determine the capability of a specific vector to transmit a virus in nature known as vector capacity (VC). The vector capacity is calculated using following formula: $\:VC=m{a}^{2}b{p}^{n}/-\text{ln}p$, and in addition to vector competence (b) includes number of females (m), daily blood feeding rate (a), probability of daily survival (p), and the extrinsic incubation period (n)^29^. To serve as a bridge vector, a mosquito species must feed on both, the amplifying host and humans. *Aedes albopictus* and *Ae. rusticus* are known to bite humans^30,31^. Besides humans as primary blood source, *Ae. albopictus* also feeds on small mammals, including rabbits, which can act as a host for TAHV. Though less studied, *Ae. rusticus* is also reported to feed on non-human mammals^30,32^. *Aedes vexans*, the only other confirmed vector for TAHV in Europe, on the other hand is known to prefer non-human mammals, but also bites humans^32^. Overall, the feeding behavior of these species makes it plausible for them to transmit TAHV from zoonotic hosts to humans. Vector competence is a crucial part of models predicting the risk of arbovirus transmission^33^. By identifying which mosquito species can transmit TAHV at which temperature and efficiency, the data can help to calculate the basic reproduction number and therefore predict risk areas.

Our study determined that *Ae. albopictus* and *Ae. rusticus* are able to transmit TAHV at a mean temperature of 27 °C. Combined with the virus’ short extrinsic incubation period of two days^26^, i.e. the time between a mosquito ingesting an infectious blood meal and the appearance of infectious virus in its saliva, it suggests that transmission could occur in Germany. In Southern Europe, mean temperatures of 27 °C are more common, which would increase the possibility of transmission in these regions^34^. *Aedes albopictus* was not able to transmit the virus at the lower tested temperature of 24 °C. Due to the limited availability of field-caught specimens, transmission of TAHV by *Ae. rusticus* could not be assessed at temperatures below 27 °C, however, future studies should prioritize investigating its transmission potential at lower temperatures, as this species is most commonly encountered in early spring, when average temperatures typically fall below 27 °C^35^.

Only two studies have investigated the vector competence of *Ae. rusticus*, showing that this species is susceptible to infection by Rift Valley fever virus (order *Bunyavirales*) and infection and transmission of West Nile virus but not Usutu virus (order *Amarillovirales*)^31,36^. Our study identified *Ae. rusticus* as a competent vector for an additional arbovirus, making TAHV only the second virus known to be transmittable by this species. *Aedes rusticus* prefers forested habitat, is widespread and native from Northern Africa through Western, and Central Europe, up to Northern Europe^37^.

Mosquitoes with infectious TAHV in their saliva exhibited two of the highest viral RNA titers in their bodies. However, some mosquitoes with similar or even higher body titers were unable to transmit the virus. Body titers of similar orders of magnitude have been reported in other vector competence studies^26,31,38,39^. However, most studies do not stratify body titers based on the infectivity of the mosquito’s saliva, limiting direct comparisons of this observation with other research. In this study, the transmission efficiency was assessed by detecting infectious virus in mosquito saliva rather than relying on viral RNA detection. While detection of viral RNA is commonly used, it does not distinguish between infectious and non-infectious viral particles, and might therefore overestimate transmission efficiency. A previous study on other arboviruses showed that viral RNA can be detected earlier and for longer in saliva, compared to infectious virus^40^. To avoid this limitation, we prioritized virus detection through cell culture.

Some limitations must be considered when interpreting the results of this study. The relatively low numbers of investigated specimens of *Ae. koreicus*,* Ae. rusticus*,* Cs. morsitans*, and *An. daciae* limits the precision of the calculated infection rates and transmission efficiencies, potentially leading to under- or overestimation of their vector competence. Statistical statements are therefore not possible for these species, however, the findings remain valuable given the current scarcity of data in this area. Screening for pre-existing TAHV infections in wild-caught mosquitoes was feasible for only two taxa (*Cx. pipiens* biotype *pipiens*, *Cx. torrentium*), therefore an overestimation of infection rate and transmission efficiency for the remaining taxa cannot be fully excluded. However, we consider this risk to be low, as TAHV is not known to circulate widely in mosquito populations in Germany. Additionally, the study was conducted under controlled laboratory conditions, which may not fully replicate the complex environmental and ecological factors influencing virus transmission in natural settings.

## Conclusion

TAHV can be transmitted at low efficiency by invasive *Ae. albopictus*, and at higher efficiency by *Ae.rusticus*, a species native to Germany and other European countries. Lower temperature reduces both the infectivity and transmission of TAHV in *Ae. albopictus*. Future studies should investigate other widespread *Aedes* species native to Germany, particularly *Ae. vexans*, *Ae. caspius*, *and Ae. sticticus*, to better understand their potential as TAHV vectors.

## Materials and methods

Mosquitoes from established laboratory colonies, field-caught adults, and field-caught eggs were used in this study. Mosquitoes were kept at 26 °C and a 70% relative humidity with a 12:12 light: dark photoperiod including 30 min of twilight. *Aedes aegypti* (Bayer company, Leverkusen, Germany) and *Anopheles stephensi* (strain SxK Nijmegen) are long established laboratory colonies. The *Ae. albopictus* colony was established in 2016/17 from eggs collected in Freiburg, Germany. Eggs of *Ae. koreicus* (Lat: 50.078411, Long: 8.251675) were collected in 2023, *Ae. j. japonicus* (Lat: 49.524, Long: 8.672) in 2023 and 2024 with ovitraps in South-West Germany. Adults of *Aedes rusticus* (Lat: 49.312851, Long: 8.301493) and *Anopheles daciae* (Lat: 49.291527, Long: 8.456074) were collected with CO_2_ baited Encephalitis Vector Survey trap (EVS trap) (BioQuip Products, Rancho Domingues, Califonia, USA) in 2024 in Southern Germany. Adults of *Cs. morsitans*/*fumipennis* were collected using an aspirator in 2024 from a forest near Oldenburg, Lower Saxony, Germany (Lat: 53.159019, Long: 8.125420). Egg rafts of *Culex torrentium* and *Culex pipiens* biotype *pipiens* were collected in the field in northern Germany (Lat: 53.467821, Long: 9.831346) in 2023. The egg rafts were reared individually and 5–10 larvae from each raft were used to identify the *Culex* species by molecular assays as described by Rudolf et al.^41^. To distinguish *An. daciae* within the *An. maculipennis* complex, it was identified to species level by molecular typing following Lühken et al.^42^. *Culiseta morsitans/fumipennis* could not be identified to species level. Therefore, the terms taxon and taxa are used when referring to this classification. All morphological identification was performed according to the key of Becker et al., 2020^43^. To exclude natural arbovirus infections in field-caught specimens, 10 randomly selected adult mosquitoes per taxon were tested using pan-Orthobunya, pan-Flavivirus, and pan-Alphavirus PCRs^44–46^. All tested specimens were confirmed negative. This screening was feasible for *Cx. pipiens* biotype *pipiens* and *Cx. torrentium*, but could not be conducted for the remaining wild-caught taxa due to limited availability of individuals.

Female mosquitoes were deprived of food for 24 to 48 h. Adult mosquitoes from laboratory colonies (*Ae. aegypti*,* Ae. albopictus*,* An. stephensi*) were aged three to 15 days, whereas adults from field-caught eggs (*Ae. koreicus*,* Ae. j. japonicus*,* Cx. torrentium*,* Cx. pipiens* biotype *pipiens*) were aged three to 18 days. The ages of field-caught adults (*Ae. rusticus*,* An. daciae*,* Cs. morsitans/fumipennis*) was unknown, however, they were infected within two to five days after capture. All experiments with live mosquitoes exposed to infectious virus were performed in an insectary of biosafety level 3 (Bernhard Nocht Institute for Tropical Medicine; Hamburg, Germany). Mosquitoes were housed in 50 mm x 100 mm cylindrical plastic tubes with corresponding plugs (Carl Roth; Karlsruhe, Germany). The mosquitoes were orally exposed to an infectious artificial blood meal, containing 50% human blood (type 0; expired blood preservations), 30% of an 8% fructose and 0.02% 4-aminobenzoic acid solution, 10% fetal bovine serum, and 10% virus stock (TAHV; Bardos et al.^3^. ; strain: Bardos92; >22 passage on BHK-J cells; stock produced: 25.07.2022; stored at -80 °C; 1.57 × 10^8^ FFU/mL), via two cotton swabs and two 50 µL drops for two hours. 4-aminobenzoic acid is an amino acid present in plants, a precursor of folic acid, and regularly used in mosquito rearing^47^, with no known interaction with viruses. The bloodmeal had a final viral titer of 1.57 × 10^7^ FFU/mL. Fully engorged females were collected and incubated at 70% relative humidity and a temperature profile of 27 °C with a ± 5 °C fluctuation over 24 h, to simulate natural temperature variance. Additionally, *Ae. albopictus* specimens were incubated at 24 ± 5 °C. These temperature profiles followed a 12 h:12 h light: dark photoperiod, with the extreme temperatures in the middle of the respective photoperiod. Hereafter, the temperature profiles 27 ± 5 °C and 24 ± 5 °C will be referred to as 27 °C and 24 °C, respectively. A solution of 8% fructose and 0.02% 4-aminobenzoic acid was available ad libitum. After 14 days of incubation, transmission capability was analyzed via salivation assay, as described by Heitmann et al.^48^. In short, the legs and wings of mosquitoes were removed while anesthetized with CO_2_. The proboscis was then inserted into a pipette tip containing phosphate-buffered saline (PBS). After 30 min, the mosquito was removed and stored at -80 °C until further analysis. The PBS/saliva mix was pipetted on BHK-J cells (*Mesocricetus auratus*, CCVL L 0179, Friedrich-Loeffler-Institute, Riems, Germany) and incubated for five days at 37 °C and 5% CO_2_. If CPE was detected, the supernatant was subjected to RNA extraction using QIAmp Viral RNA Mini Kit (Qiagen; Hilden, Germany), according to the manufacturer’s instructions. If possible, 30 mosquito specimens were tested per experimental condition for infection and transmission; otherwise, all available specimens were included. Examining a minimum of 30 specimens per condition is a widely accepted standard in vector competence studies, enabling the detection of transmission efficiencies (TE) as low as 3%. This sample size allows for the identification of biologically meaningful vector competence (TE ≥ 3%) while keeping experimental effort manageable^39^. The mosquito bodies, excluding wings and legs, were homogenized in 500 µL Dulbecco’s Modified Eagle Medium, using a Bio-Vortexer (Biospec Products; Bartlesville, USA). Subsequently, the supernatant was subjected to RNA extraction using MagMAX CORE nucleic acid purification kit (Applied Biosystems, Thermo Fisher Scientific; Waltham, USA), according to the manufacturer’s instructions.

Both the RNA from the supernatant of the cell cultures inoculated with saliva that showed cytopathic effect, and from the bodies, were tested for the presence of TAHV RNA, M-segment, via qRT-PCR using QuantiTect probe RT-PCR kit (Qiagen; Hilden, Germany), and VetMAX™ Xeno™ Internal PositiveControl (Applied Biosystems, Thermo Fisher Scientific; Waltham, USA). Reactions were performed in 20 µL reaction volume containing 1x reaction buffer, 1x RT-PCR mix, 1 µM forward primer (TGCTGGGAAACAGAATTTACTGAG), 1 µM reverse primer (TGGTGACTGTACATTCTCCTGAG), 0.2 µM probe (CY5-TCCTGCAACTCCTTACCCCATCAC-BBQ650), 1x Xeno™ VIC™ primer probe mix, 20,000 copies Xeno™ IPC RNA, and 2 µL template. The PCR conditions were as follows: 20 min 50 °C, 15 min 95 °C, and 45 cycles of 5 Sect. 95 °C and 60 Sect. 60 °C. A synthetic oligonucleotide (ATGTGCTGGGAAAC AGAATTTACTGAGTACA TCCAATTTAAACAGAGT GATEGGGTAAGGAGTTG CAGGATGAAAG ACTCAGGAGAAT GTACAGTCACCATCA) was used as a positive control and for quantification of the body titer. Samples were considered positive if a CY5 signal was detected and the calculated total body titer was above the below-listed limit of detection. Samples were considered negative when no CY5 signal but a positive VIC signal was detected.

The RT-qPCR was validated according to the “Minimum Information for Publication of Quantitative Real-Time PCR Experiments” guidelines set by Bustin et al.^49^. A ten-fold dilution series of the TAHV standard, ranging from 0.18 to 1.8 × 10^8^ copies/µL was analyzed in five replicates using the specified RT-qPCR protocol. Following performance indicators were determined: limit of detection: 4.05 × 10^5^ copies/mosquito body, with a standard deviation of 0.759 Cqs; linear dynamic range: detection limit to ≥ 4.05 × 10^10^ copies/mosquito body (i.e. the fluorescence signal is in a linear proportion to the concentration in this range). The calibration curve had a coefficient of determination of 0.9962 with a slope of -3.351, and a y-intercept of 54.86. The PCR efficiency was 0.9906, i.e., 99.1% of the target molecules were amplified per cycle.

To determine statistically significant differences, statistical tests were performed using GraphPad Prism (version 10.3.1 for Windows, GraphPad Software, www.graphpad.com). A p-value less than 0.05 was considered significant.

## Data Availability

The datasets generated during and/or analyzed during the current study are available from the corresponding author on request.
